# Microbial Mastery in Food Innovation: Synergizing Flavor, Functionality, and Safety for Next-Generation Nutrition

**DOI:** 10.3390/foods14173081

**Published:** 2025-09-01

**Authors:** Xiaoyan Liu, Guangsen Fan

**Affiliations:** School of Food and Health, Beijing Technology and Business University, Beijing 100048, China; liuxiaoyan8112@163.com

The contemporary food landscape is defined by a critical imperative: to develop sustainable, nutritious, and sensorially appealing products that meet evolving consumer demands for naturalness and health benefits. Within this context, the strategic application of fermentation and enzymatic bioprocessing emerges as a cornerstone technology, uniquely positioned to bridge the gap between nutritional functionality, safety assurance, and consumer acceptability. This editorial synthesizes pivotal insights from a compelling suite of studies, collectively demonstrating the transformative power of microbial and enzymatic interventions in redefining food matrices, enhancing sensory profiles, mitigating risks, and unlocking bioactive potential across diverse food categories.

The challenge of undesirable sensory attributes, particularly in novel and sustainable protein sources, finds potent solutions through the action of lactic acid bacteria (LAB) and yeast [1]. Off-flavors characterized as “green,” “beany,” or “fatty,” frequently arising from aldehydes, ketones, and pyrazines [2,3], constitute significant barriers to consumer adoption. Compelling evidence exists for LAB fermentation’s efficacy in modulating the volatile profile of Chlorella vulgaris. Specific strains, notably *Lcb. Paracasei* UPCCO 2333, achieved significant reductions in key off-flavor compounds like hexanal, aldehydes, ketones, pyrazines, and terpenes, while concurrently promoting the synthesis of pleasant esters, thereby enhancing the overall aroma profile of this nutritionally dense microalga. Parallel advancements are evident in plant proteins. Fermentation of soybean protein hydrolysates using the synergistic combination of *Tetragenococcus halophilus* (LAB) and *Zygosaccharomyces rouxii* (yeast) resulted in a marked decrease in aldehydes and ketones responsible for characteristic “beany” notes. This reduction was accompanied by a significant enrichment in desirable volatile compounds, including alcohols, esters, acids, and sulfurs, crucial for complex and appealing flavor profiles. Importantly, the study highlighted the nuanced impact of inoculation strategy, with sequential inoculation proving superior to simultaneous co-inoculation for flavor optimization. These findings showed that microbial metabolism is a primary driver shaping the volatile organic compound (VOC) profiles essential for sensory quality [4,5].

Beyond the realm of sensory enhancement, targeted bioprocessing offers robust pathways to optimize nutritional functionality and ensure microbiological safety. In the burgeoning plant-based sector, the efficacy of a specific LAB consortium (*Lactiplantibacillus plantarum* 82 and *Leuc. carnosum* 4010) in fermenting nut-based matrices was demonstrated. This combination induced rapid acidification (achieving pH < 4.4 within 18 h), effectively controlling challenging pathogens such as *Listeria monocytogenes* (eliminated within seven days) and *Salmonella enterica serovar Enteritidis.* Crucially, this fermentation concurrently enhanced the functional profile of the product, increasing phenolic content and generating potent antimicrobial metabolites like phenyllactic acid and hydroxyphenyllactic acid, while also developing desirable “cheese-like” aroma compounds such as acetoin and diacetyl. Enzymatic strategies provide complementary power to microbial action, as illustrated by the innovative development of a low-calorie, lactose-free, brown fermented milk. Utilizing low-temperature lactase hydrolysis, this process achieved a remarkable 33-fold reduction in lactose content (to 0.06 g/100 g), minimized the formation of potentially harmful Maillard reaction by-products like 5-hydroxymethylfurfural (5-HMF) and 3-deoxyglucosone by over 20%, and improved protein composition and product stability (notably water-holding capacity and suspension stability over 28 days storage). This approach directly addresses the significant challenge of sugar reduction in appealing dairy formats. Furthermore, the valorization of agricultural by-products is exemplified by the biological purification of soy molasses using the yeast *Wickerhamomyces anomalus* YT312. This strain demonstrated an exceptional capability under optimized conditions to selectively and completely remove sucrose while preserving over 90% of the functional oligosaccharides raffinose and stachyose, thereby significantly enhancing the prebiotic value and functionality of this underutilized stream.

The final quality and functionality of fermented foods are profoundly influenced by the complex interplay between the inherent microbial community structure, the composition of the substrate, and the specific processing parameters employed. This interplay was explored using defatted wheat germ (DWG), a nutritionally rich but structurally challenging by-product. Air classification yielded distinct high-fiber (HF) and high-protein/low-fiber (LF) fractions. Subsequent bioprocessing—combining enzymatic treatment (xylanase) with tailored LAB fermentation—dramatically increased free amino acid content (up to 6 g/kg) and significantly improved protein digestibility (80% vs. 63% in control bread). Incorporating the bioprocessed HF fraction into bread formulations successfully elevated fiber content beyond 3.6 g/100 g (qualifying as a “source of fiber”), lowered the glycemic index (84 vs. 89), and improved technological properties like crumb porosity, elasticity, and crust color. Similarly, modifying traditional fermentation starters can yield significant flavor advantages. Introducing shiitake mushroom (*Lentinula edodes*) into Jiuqu, the essential starter for Chinese rice wine (Huangjiu), induced a significant shift in microbial ecology. High-throughput sequencing revealed *Aspergillus*, *Lactobacillus*, and *Vibrio* demonstrated strong positive correlations with key flavor compounds unique to shiitake mushrooms, highlighting the potential for directed microbial community design.

The intricate and often defining relationship between microbiota and volatile compound generation is further underscored in studies on traditional fermented beverages. Analysis of Chinese sweet rice wine (CSRW) starters using high-throughput sequencing identified core functional microbial genera (*Weissella, Pediococcus, Lactobacillus, Saccharomycopsis, Rhizopus, Wickerhamomyces, Cyberlindnera*). Spearman correlation analysis revealed nineteen significant positive correlations between fifteen key volatile compounds (alcohols, acids, aldehydes, esters) and these nine microbial genera. Notably, *Weissella* and *Saccharomycopsis* strongly correlated with phenylethyl alcohol (a key aroma compound), while *Pediococcus* correlated with 2-nonanol. This complex microbial–volatile network fundamentally dictates the characteristic regional profiles of CSRW. During black tea fermentation, dynamic shifts in microbial communities, particularly the dominance of bacteria (e.g., *Chryseobacterium* and *Sphingomonas*) and fungi (e.g., *Pleosporales*), were elucidated. These shifts were linked to increased activity in key metabolic pathways (glycolysis, pyruvate dehydrogenase, TCA cycle) and the consequential accumulation of critical quality determinants: amino acids, soluble sugars, and tea pigments. Correlation analysis further linked specific bacterial abundances to the content of tea polyphenols and catechins. These studies showed that the health benefits of fermented foods are intrinsically linked to the metabolic activities of complex microbial communities [6], and extend this principle to flavor development, demonstrating that the precise manipulation of consortia and processing conditions allows for tailored functional and sensory outputs [7].

The breadth of research presented herein illuminates a coherent and promising trajectory for the future of food innovation. Collectively, these studies demonstrate that the intelligent application of fermentation and enzymatic bioprocessing can simultaneously achieve multiple critical objectives: systematically reduce or eliminate objectionable off-flavors while developing complex, desirable aroma profiles in challenging matrices like microalgae, plant proteins, and agricultural by-products; significantly increase the bioavailability and concentration of bioactive compounds (free amino acids, phenolics, functional oligosaccharides, and SCFA precursors), improve macronutrient digestibility, reduce glycemic impact, and generate beneficial metabolites with health-promoting potential; leverage rapid acidification and the production of specific antimicrobial metabolites (organic acids, bacteriocins, and phenyllactic acid) for the effective control of foodborne pathogens; and transform underutilized or low-value agricultural by-products (e.g., DWG, soy molasses) into high-value, functional food ingredients, contributing to a more circular and sustainable food system.

To fully realize this potential, future research must prioritize several key areas. Deeper mechanistic insights into microbial interactions, metabolic fluxes, and the specific pathways leading to desired flavor and bioactive compounds are essential, necessitating the application of integrated multi-omics approaches (genomics, transcriptomics, metabolomics) [8]. Translating lab-validated processes to industrial scale requires rigorous optimization for cost-effectiveness, scalability, and robustness under production conditions [9]. Finally, sensory science must be integrated throughout the product development lifecycle to ensure that improvements in nutrition, sustainability, and safety are matched by high levels of consumer acceptability and hedonic appeal [10]. As highlighted in the broader context of flavor and food bioactive research, the convergence of microbiology, enzymology, process engineering, and sensory science [11], augmented by emerging technologies like artificial intelligence for the predictive modeling of fermentation outcomes and microbial interactions [12], and nanotechnology for the targeted delivery and controlled release of bioactives and flavors [13], holds the definitive key to delivering the next generation of foods. These foods must be truly nutritious, sensorially delightful, inherently safe, and produced sustainably. The compelling evidence within this collection unequivocally positions the precise harnessing of microbial power as central to achieving this future.
